# A Tutorial on Auditory Attention Identification Methods

**DOI:** 10.3389/fnins.2019.00153

**Published:** 2019-03-19

**Authors:** Emina Alickovic, Thomas Lunner, Fredrik Gustafsson, Lennart Ljung

**Affiliations:** ^1^Department of Electrical Engineering, Linkoping University, Linkoping, Sweden; ^2^Eriksholm Research Centre, Oticon A/S, Snekkersten, Denmark; ^3^Hearing Systems, Department of Health Technology, Technical University of Denmark, Lyngby, Denmark; ^4^Swedish Institute for Disability Research, Linnaeus Centre HEAD, Linkoping University, Linkoping, Sweden

**Keywords:** cocktail-party problem, auditory attention, linear models, stimulus reconstruction, canonical correlation anaysis (CCA), decoding, encoding, sparse representation

## Abstract

Auditory attention identification methods attempt to identify the sound source of a listener's interest by analyzing measurements of electrophysiological data. We present a tutorial on the numerous techniques that have been developed in recent decades, and we present an overview of current trends in multivariate correlation-based and model-based learning frameworks. The focus is on the use of linear relations between electrophysiological and audio data. The way in which these relations are computed differs. For example, canonical correlation analysis (CCA) finds a linear subset of electrophysiological data that best correlates to audio data and a similar subset of audio data that best correlates to electrophysiological data. Model-based (encoding and decoding) approaches focus on either of these two sets. We investigate the similarities and differences between these linear model philosophies. We focus on (1) correlation-based approaches (CCA), (2) encoding/decoding models based on dense estimation, and (3) (adaptive) encoding/decoding models based on sparse estimation. The specific focus is on sparsity-driven adaptive encoding models and comparing the methodology in state-of-the-art models found in the auditory literature. Furthermore, we outline the main signal processing pipeline for how to identify the attended sound source in a cocktail party environment from the raw electrophysiological data with all the necessary steps, complemented with the necessary MATLAB code and the relevant references for each step. Our main aim is to compare the methodology of the available methods, and provide numerical illustrations to some of them to get a feeling for their potential. A thorough performance comparison is outside the scope of this tutorial.

## 1. Introduction

The first use of the term *cocktail party* in the context of auditory scene analysis appeared in Cherry (1953), where it was used to refer to the challenge of focusing on a single sound source, often a speech stream, while suppressing other unwanted sounds in a noisy and complex background. The ability to segregate and follow a sound source of interest in a cocktail party environment is one of the hallmarks of brain functions. Although this is a highly ill-posed problem in a mathematical sense, the human brain instantly solves this problem, with a compelling ease and accuracy that is difficult to be matched by any currently available algorithm. However, recent studies have shown the potential of model-based algorithms to assist intelligent hearing aids, and the purpose of this tutorial is to provide a rather broad coverage of the mathematical tools available for solving the cocktail party problem. The algorithms are illustrated on examples from datasets previously used in several studies. The algorithms in this tutorial are relatively simple and computationally inexpensive, although further research on algorithm optimization is needed to achieve real-time performance.

Neural networks and cognitive processes assist the brain in parsing information from the environment (Bregman, 1994). These processes allow us to perform everyday tasks with remarkable ease and accuracy, for example, enjoying our time with friends in crowded places such as restaurants and cafes while being alert to salient sound events such as someone calling our name. The intrinsic complexity of the background is hidden by the brain's process of perceiving and selectively attending to any sound source: (a) competing acoustic sources (stimuli) emit acoustic signals and (b) are subsequently mixed, (c) the mixture of incoming sound streams enters the ear(s), (d) this mixture is resolved such that (e) the attended sound is perceived, and (f) the remaining, unwanted streams of sound are effectively attenuated within the human auditory cortex.

There are many studies on deciphering human auditory attention. The majority of these studies have generally focused on brain oscillations (Obleser and Weisz, 2011; Weisz et al., 2011; Henry et al., 2014) and speech entrainment (Ding and Simon, 2012a,b; Mesgarani and Chang, 2012; Pasley et al., 2012; Mirkovic et al., 2015; O'Sullivan et al., 2015, 2017; Ekin et al., 2016; Biesmans et al., 2017; Fuglsang et al., 2017; Kaya and Elhilali, 2017; Van Eyndhoven et al., 2017; Haghighi et al., 2018) in electroencephalography. Broadly speaking, the two most common approaches in the development of speech (envelope) entrainment are (1) *encoding*, i.e., estimating the neural responses from the sound features, and (2) *decoding*, i.e., estimating the sound from the neural response features. In most of these studies, the linear filters are computed using “dense” least-squares (LS) optimization tools. However, it is also possible to exploit an alternative approach based on sparse estimation. Sparse estimation has shown great potential in diverse signal processing applications (Sepulcre et al., 2013; Akram et al., 2016, 2017; Rao et al., 2016; Miran et al., 2018).

As a further alternative to encoding and decoding, *bidirectional* hybrid approaches (Dmochowski et al., 2017; de Cheveigné et al., 2018), such as *canonical correlation analysis (CCA)*, aim to combine the strengths (and weaknesses) of encoding and decoding methods. A recent work (de Cheveigné et al., 2018) supports the view that CCA-based classifier schemes may provide higher classification performance compared to encoding and decoding methods.

The applications of attention deciphering are diverse, including robotics, brain-computer interface (BCI), and hearing applications (see e.g., Li and Wu, 2009; Lunner and Gustafsson, 2013; Gao et al., 2014; Khong et al., 2014; Lunner, 2015; Tsiami et al., 2016). In fact, there is currently increased interest in auditory attention identification in, for instance, the hearing aid industry. The reason for this interest is that for a hearing-impaired listener, the ability to selectively attend to a desired speaker in a cocktail party situation is highly challenging. With an aging population with an increasing number of hearing-impaired individuals, increased understanding of the underlying mechanisms of the cocktail party problem is highly needed. Along the same lines, the hearing aid companies are also interested in applying auditory attention deciphering (AAD) techniques for cognitive control of a hearing aid and its noise-reduction algorithms (Das et al., 2017; Van Eyndhoven et al., 2017).

However, despite the increasing interest in this problem from the audiology and neuroscience research communities (Fritz et al., 2007; Mesgarani and Chang, 2012; Jääskeläinen and Ahveninen, 2014; Kaya and Elhilali, 2017), the basis for the computational models of the brain's ability to selectively attend to different sound sources remains unclear.

The primary objective of this study is to explain how to use linear models and identify a model with sufficiently high performance in terms of attention deciphering accuracy rates and computational time. Our ultimate goal is to provide an overview of the state-of-the-art for how linear models are used in the literature to *decipher human auditory attention* by exploiting the *brain activity* elicited during attentive listening to a single sound source in an acoustically complex background.

This contribution focuses on the classification of auditory attention by using multivariate linear models. Consequently, we do not cover other aspects of auditory attention and scene analysis, and to limit the scope, we do not cover (computational) auditory scene analysis (CASA) (Wang and Brown, 2006; Wang et al., 2009; Snyder et al., 2012; Gutschalk and Dykstra, 2014; Alain and Bernstein, 2015; Simon, 2017), auditory attention modeling (Kaya and Elhilali, 2017), speech masking (Scott and McGettigan, 2013; Evans et al., 2016), and sound segregation and localization (Ahveninen et al., 2014; Middlebrooks, 2017).

An important note regarding the current auditory attention identification methods is that these methods require access to the clean speech signals, which are usually not available in practice. CASA methods are then necessary to provide these. Recent attempts to perform attention deciphering without access to the individual speakers (but noisy speech mixtures instead) may provide a useful way to approach solving this problem. The study of S. Van Eyndhoven (Van Eyndhoven et al., 2017), later improved by Das Das et al. (2017), was the first that tackled this problem, based on beamforming methods. O'Sullivan later also did a similar study, using deep learning (O'Sullivan et al., 2017). After separating the individual speakers in the mixture, these studies used the linear models discussed in this tutorial to identify the sound source of a listener's interest.

The outline of this contribution is as follows. To obtain accurate attention deciphering using EEG (electroencephalography) / MEG (magnetoencephalography) sensors, several important factors need to be considered. First, the algorithms that are currently used to identify the attended sound source need to be accurately described, which is the topic of section 2. Note that we must always first preprocess the data to avoid problems in the later encoding/decoding procedures, which is also a topic of section 2. Based on the analysis of the models in section 2, we can construct different models. In section 3, we discuss the datasets used in this contribution to study different auditory attention identification methods. The practical implementation of the discussed algorithms is the topic of section 4, where we provide experimental results for some different examples and datasets. We end this contribution with some concluding remarks and (potential) future improvements in section 5.

## 2. Linear Models for Auditory Attention Deciphering

In this section, we explain the basics of linear modeling. Furthermore, we introduce some of the concepts from machine learning (ML) that are frequently used in the auditory attention identification literature. The last decade has witnessed a large number of impressive ML applications that involve large amounts of data, and our application of audio-EEG data is one area that has thus far remained rather unexplored. The subject of designing the linear models is introduced in section 2.1. How to select the model is a crucial part of any estimation problem. Thus, we discuss different modeling approaches in sections 2.3–2.4.

### 2.1. The Sound and EEG Signals

We assume that at any given point in space, a time-varying sound pressure exists that originates from *n*_*u*_ sound streams *p*_*i*_(*t*), *i* = 1, 2, …, *n*_*u*_, emitted by one or more sound sources (e.g., individual talkers and loudspeakers). The resulting sound pressure can be conceptually written as a sum

(1) $$\begin{array}{l} p\left(t\right)=\overset{n_{u}}{\underset{i=1}{\sum }}p_{i}\left(t\right). \end{array}$$

This mixture is what the ear decodes and what can be sampled by a microphone. The latter results in a discrete time signal *p*[*k*] = *p*(*kT*_*s*_), where *T*_*s*_ is the sampling interval, which typically corresponds to a sampling frequency of $f_{s}^{p}=1/T_{s}=44100$ Hz.

The EEG signals are sampled by *n*_*y*_ EEG electrodes denoted *y*_*j*_[*k*], *j* = 1, 2, …, *n*_*y*_. The EEG sampling frequency $f_{s}^{y}$ is considerably smaller than the sampling frequency of the sound $f_{s}^{p}$. Typical values in experiments in this field are *n*_*u*_ = 2, *n*_*y*_ = {64, 128} and $f_{s}^{y}=512$ Hz. To synchronize the data streams to the same sampling frequency, the ratio $f_{s}^{p}/f_{s}^{y}$ defines a decimation factor that is needed to reduce the sampling rate of the sound. This downsampling needs to be done only after the envelope extraction of the individual sound sources *p*_*i*_(*t*). In the following paragraphs we will describe each of these steps in more detail.

Next, we present the basic steps that are commonly used in practice in this application:

- Extract the envelope of the audio signal, which can be performed in several ways. A complete overview of the envelope extraction methods for AAD is presented in Biesmans et al. (2017). The resulting sound signal will be denoted *u*[*k*], which in the literature is supposed to be the sum $u\left[k\right]=\overset{n_{u}}{\underset{i=1}{\sum }}u_{i}\left[k\right]$ of *n*_*u*_ envelopes *u*_*i*_[*k*], but it should be noted that *u*[*k*] will never be used in practice as the access to the individual sound streams *u*_*i*_[*k*] is needed when applying AAD techniques. Speech envelopes are spectrotemporally sparse, and therefore the equation is approximately true enough for the purposes used here.
- Downsample the EEG signal and the audio signals to the same sampling rate (e.g., to 64 Hz), which can be performed using the *nt_dsample* function from the NoiseTools toolbox (http://audition.ens.fr/adc/NoiseTools/) (Yang et al., 1992; Ru, 2001) or MATLAB built-in downsampling methods, such as *decimate* or *resample* functions.
- Bandpass filter both the EEG and the sound signals using a bandpass filter between 1 and 8 Hz, which is the frequency interval where the brain processes auditory information (Zion Golumbic et al., 2013).

The following code performs this operation, as was proposed in O'Sullivan et al. (2015):


p     = resample(p,44096,44100);
     % Resample to a multiple of 64 Hz
pc    = hilbert(p);
     % Transform from real to analytic signal
u     = decimate(abs(pc),44096/64);
     % Downsampling to 64 Hz, including an
     anti-alias
    anti-alias
[b,a] = butter(3,[2 8]/64^*^2);
    % Bandpass filter with passband [2,8] Hz
uf    = filter(b,a,u);
    % Causal filtering to keep causality


Without loss of generality, we will assume that the attended sound source is *u*_1_[*k*], while the other sources, *u*_*i*_[*k*] for *i* > 1, represent nuisance sound sources.

### 2.2. Data Notation

We denote all scalars by lowercase letters, e.g., *w*, and all vectors and matrices by uppercase letters, e.g., *W*, unless stated otherwise. The (*p, q*) entry, *p*−th row and *q*−th column in *W* are expressed as [*W*]_*p,q*_, *W*_*p*, :_ and *W*_:, *q*_, respectively, and the *p*−th entry in vector *U* is expressed as *U*_*p*_. The transpose of the matrix *W* is denoted as *W*^*T*^. The functions ∥*W*∥_*F*_ (Frobenius norm) and ∥*U*∥_2_ (Euclidean or *l*_2_ norm) return the matrix-valued norm and vector-valued norms, respectively, and $∥W{∥}_{F}^{2}=\text{trace}\left({\text{W}}^{\text{T}}\text{W}\right)\text{and}∥\text{U}{∥}_{2}^{2}={\text{U}}^{\text{T}}\text{U}$. The *l*_1_ penalty term is defined as $∥W{∥}_{1}=\underset{p,q}{\sum }|{\left[W\right]}_{p,q}|$. The letter *n* with an index will denote the dimension of a vector, for instance, *n*_*y*_ and *n*_*u*_, as previously introduced.

To have a compact notation avoiding one or more indices, we will summarize the data in the data vectors *U*_*i*_ and *Y*_*j*_, the data matrices *U* and *Y*, which are defined as follows:

(2) $$\begin{array}{l} {\left[Y_{j}\right]}_{k}=y_{j}[k],\;k=1,\ldots ,N,\;j=1,2,\ldots ,n_{y}, \end{array}$$

(3) $$\begin{array}{l} {\left[Y\right]}_{kj}\;=y_{j}[k],\;k=1,\ldots ,N,\;j=1,2,\ldots ,n_{y}, \end{array}$$

and similarly for *U* and *U*_*i*_.

For a model that takes the latest *n*_*a*_ data points into account, we define the Hankel matrix

(4) $$\begin{array}{l} {\left[H(Y_{j})\right]}_{kn}=y_{j}[n_{a}+k-n],k=1,\ldots ,N-n_{a}+1,\\ \;n=1,2,\ldots ,n_{a}, \end{array}$$

and similarly for $H\left(U_{i}\right)$. We will refer to the data as *Y*_*j*_, *U*_*i*_, *Y, U*.

### 2.3. Correlation-Based Learning

Correlation-based learning aims to find the pattern in the EEG signal that best correlates to the target sound *u*_1_(*t*) with less correlation to the distracting sounds *u*_*i*_(*t*), *i* ≠ 1. Typical correlation-based learning approaches are:

1. Cross-correlation:
  - Zero-lag cross-correlation: The normalized covariance between each speech signal *U*_*i*_ and each EEG signal *Y*_*j*_, i.e., $c_{ij}=\frac{Cov\left(U_{i},Y_{j}\right)}{\sqrt{Var\left(U_{i}\right)Var\left(Y_{j}\right)}}$. The drawback with zero-lag cross-correlation is that it assumes that both *U*_*i*_ and *Y*_*j*_ are synchronized in time, which is hardly the case.
  - Time-lag cross-correlation: Here one of the sequences is delayed (time-lagged) before the correlation is computed. There is here one extra degree of freedom, so one has to maximize cross-correlation with respect to this lag.
2. Canonical Correlation Analysis (CCA).

The disadvantage of correlation-based approaches is that they compare sample by sample for the entire batch and are thus less effective if there is a dynamical relationship between *U* and *Y*, in which case only a few samples around the current time would exhibit a significant correlation. CCA corresponds to a linear model of the whole segment of speech, and the model is by construction non-causal. The segment length is an important design parameter corresponding to the model order in FIR models.

### 2.4. Linear Models

The linear filter formalism we use is based on the shift operator *q* defined by *q*^−*n*^*x*[*k*] = *x*[*k* − *n*] and *q*^*n*^*x*[*k*] = *x*[*k* + *n*] for all *n*. A causal FIR filter can then be written as

(5) $$\begin{array}{l} \begin{array}{c} y_{j}\left[k\right]=B_{i}\left(q\right)u_{i}\left[k\right]=\left(b_{i0}+b_{i1}q^{-1}+\cdots +b_{in_{b}}q^{-n_{b}}\right)u_{i}\left[k\right]\\ =b_{i0}u_{i}\left[k\right]+b_{i1}u_{i}\left[k-1\right]+\cdots +b_{in_{b}}u_{i}\left[k-n_{b}\right]. \end{array} \end{array}$$

Similarly, an IIR filter can be written as

(6) $$\begin{array}{l} A_{j}\left(q\right)y_{j}\left[k\right]=B_{i}\left(q\right)u_{i}\left[k\right],\\ \left(1+a_{j1}q^{-1}+\cdots +a_{jn_{a}}q^{-n_{a}}\right)y_{j}\left[k\right]=y_{j}\left[k\right]+a_{j1}\\ \;y_{j}\left[k-1\right]+\cdots +a_{jn_{a}}y_{j}\left[k-n_{a}\right]=B_{i}\left(q\right)u_{i}\left[k\right],\\ y_{j}\left[k\right]=-a_{j1}y_{j}\left[k-1\right]-\cdots -a_{jn_{a}}y_{j}\left[k-n_{a}\right]+B_{i}\left(q\right)u_{i}\left[k\right]. \end{array}$$

It should also be noted that (6) does not represent the general form of *A*_*j*_(*q*), i.e., the filter *A*_*j*_(*q*) can be generalized so that positive exponents can also be used for *q*, as explained in the remainder of this section.

Implementation requires stability. The IIR filter specified by *A*_*j*_(*q*) can be causally stably implemented *forward* in time only if all roots to the polynomial *A*_*j*_(*q*) are *inside* the unit circle. We denote such a filter with *A*^*f*^(*q*). Conversely, a filter with all roots *outside* the unit circle can be anti-causally implemented in a stable way *backward* in time, and we denote such a filter with *A*^*b*^(*q*). Any IIR filter can be split into two parts with one causal and one anti-causal part. For more details on these issues, see basic text books in signal processing, for instance (Gustafsson et al., 2010).

Given this brief background, there are two fundamentally different ways to define a model for listening attention, forward or backward in time,

(7) $$\begin{array}{l} y_{j}\left[k\right]=\overset{n_{u}}{\underset{i=1}{\sum }}\frac{B_{i}^{f}\left(q\right)}{A_{j}^{f}\left(q\right)}u_{i}\left[k\right]+e{\;}_{j}^{f}\left[k\right] \end{array}$$

(8) $$\begin{array}{l} u_{i}\left[k\right]=\overset{n_{y}}{\underset{j=1}{\sum }}\frac{A_{j}^{b}\left(q\right)}{B_{i}^{b}\left(q\right)}y_{j}\left[k\right]+e_{i}^{b}\left[k\right] \end{array}$$

The first model corresponds to the forward model (using superscript *f* for forward), where each EEG signal is explained as a sum of filtered sound signals plus additive noise to account for measurement errors and model imperfections, while the other model corresponds to the inverse backward model (denoted with superscript *b*). Another note, positive exponents are used for *q* in backward models. It is assumed that both filters are causally stable, implying that $A_{j}^{f}$ and $B_{i}^{b}$ are polynomials with all roots inside the unit circle. The roots of $B_{j}^{f}$ and $A_{i}^{b}$ can be both inside and outside the unit circle generally. This means that inverting the forward model does not give a causally stable backward model, and is thus not in general a valid backward model. In other words, the models are not identical or related in simple terms. Also the noise realizations $e_{j}^{f}\left[k\right]$ and $e_{i}^{b}\left[k\right]$ are different and can have quite different characteristics.

Note, however, that one can mix a forward and backward model in a non-causal filter. Combining both model structures gives the linear filter

(9) $$\begin{array}{l} y_{j}\left[k\right]=\overset{n_{u}}{\underset{i=1}{\sum }}\left(\frac{B_{i}^{f}\left(q\right)}{A_{j}^{f}\left(q\right)}+\frac{B_{i}^{b}\left(q\right)}{A_{j}^{b}\left(q\right)}\right)u_{i}\left[k\right]+e_{j}\left[k\right], \end{array}$$

and similarly for the backward model. This can be seen as a non-causal filter with poles both outside and inside the unit circle.

Given such a linear filter, one can reproduce an estimate ŷ_*j*_[*k*] of the EEG signal. For instance, the causally stable part can be implemented with


for j=1:ny
   yijhat[:,j]=filter(bf(j,:),af(i,:),U(:,i));
end
yihat=sum(yijhat,2);


Here, af denotes the matrix of polynomial coefficients for the polynomials $A_{i}^{f}\left(q\right)$ and so forth. A good model should provide a small estimation error *y*_*j*_[*k*] − ŷ_*j*_[*k*]. We will return to the issue of parameter estimation, or system identification (Ljung, 1998), shortly, but note that there is no good model in the traditional sense. All linear models share the property that the prediction errors are of the same order as the signal itself. In other words, the least squares loss function will be only somewhat smaller than the sum of squared measurements, which would be the least squares loss function for the trivial signal predictor ŷ_*j*_[*k*] = 0 for all times *k* and all channels *j*.

The use of IIR (infinite impulse response) models is still unexplored in this area; thus, we will restrict the discussion to FIR (finite impulse responses) models, having denominators $A_{j}^{f}\left(q\right)=1$ in (7) and $B_{i}^{b}\left(q\right)=1$ in (8) equal to unity, in the following.

### 2.5. FIR Models for Encoding and Decoding

Here, we explain two modeling perspectives that are widely used in auditory research: *forward* and *inverse* (backward) modeling. Encoding and decoding are two special cases of supervised learning of forward and backward models, respectively (Haufe et al., 2014). The encoding and decoding models applied in cognitive electrophysiology are described in greater detail in Holdgraf et al. (2017). The traditional encoding approach attempts to predict neural responses (EEG) given the *sound stimulus*

(10) $$\begin{array}{l} y_{j}\left[k\right]=B_{i}^{f\left(q\right)}u_{i}\left[k\right]+e_{j}^{f}\left[k\right]\;\left(\text{encoding}\right) \end{array}$$

Note that there is one filter $B_{i}^{\left(q\right)}$ for each input and output combination. Here, ${ŷ}_{j}\left[k\right]=B_{i}^{f\left(q\right)}u_{i}\left[k\right]$ will be referred to as a neural prediction.

In contrast, the decoding approach attempts to extract the sound from the neural responses (EEG)

(11) $$\begin{array}{l} u_{i}\left[k\right]=\overset{n_{y}}{\underset{j=1}{\sum }}A_{j}^{b}\left(q\right)y_{j}\left[k\right]+e_{i}^{b}\left[k\right]\;\left(\text{decoding}\right) \end{array}$$

**Figure 1:**
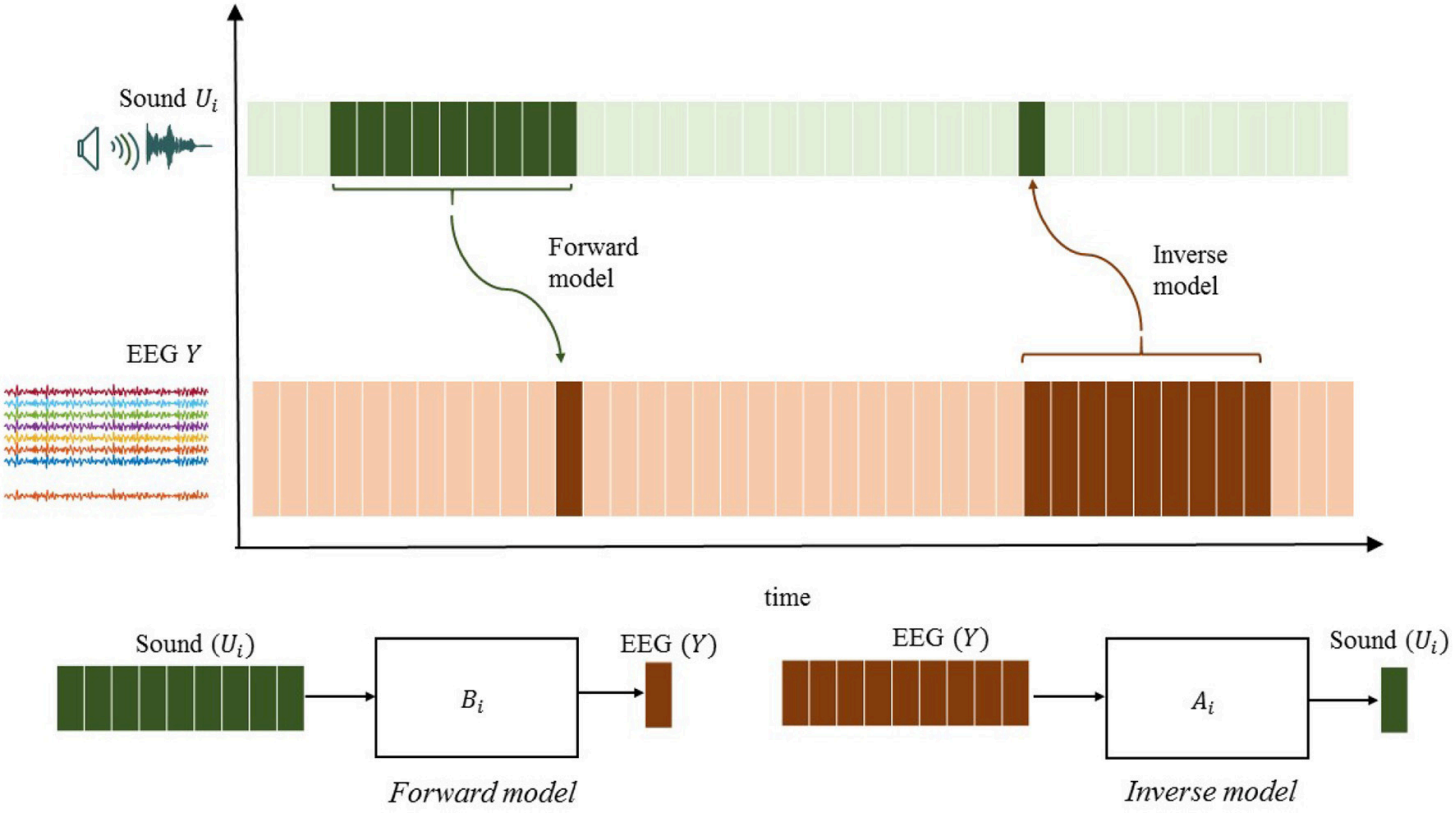
Illustration of the essential difference between encoding and decoding methods.

Similarly, ${û}_{i}\left[k\right]=\overset{n_{y}}{\underset{j=1}{\sum }}A_{j}^{b}\left(q\right)y_{j}\left[k\right]$ will be referred to as a reconstructed stimulus. Note that û_*i*_[*k*] usually captures the neural responses *y*_*j*_[*k*] after stimuli presentation at time step *k*. The stimulus reconstruction (SR) approach, which has received the greatest attention in the auditory literature, compares the reconstructed sound waveform with the actual waveform to make a decision on the attended sound source. Figure 1 illustrates the difference between the encoding and decoding approaches.

### 2.6. Parameter Estimation

The encoding and decoding models (10)–(11) can be more conveniently written in matrix-vector form as

(12) $$\begin{array}{l} Y_{j}=H\left(U_{i}\right)B_{i}^{f}+E_{j}^{f}, \end{array}$$

(13) $$\begin{array}{l} U_{i}=\underset{j}{\sum }H\left(Y_{j}\right)A_{j}^{b}+E_{i}^{b}, \end{array}$$

using the Hankel matrices defined in (4), and $B_{i}^{f}$ and $A_{j}^{b}$ are the vectors consisting of the coefficients of the polynomials $B_{i}^{f\left(q\right)}$ defined in (5) and $A_{j}^{f}\left(q\right)$ defined in (6), respectively.

The model in (12) defines an estimation error

(14) $$\begin{array}{l} \epsilon_{j}=Y_{j}-H\left(U_{i}\right)B_{i}^{f}, \end{array}$$

from which one can define an LS loss function

(15) $$\begin{array}{l} W\left(B_{i}^{f}\right)=\|Y_{j}-H\left(U_{i}\right)B_{i}^{f}{\|}_{2}^{2}. \end{array}$$

This loss function defines a quadratic function in the parameters *B*_*i*_. Minimization provides the LS estimate as

(16) $$\begin{array}{l} {\hat{B}}_{i}^{f}=\underset{B_{i}^{f}}{\mathrm{argmin}}W\left(B_{i}^{f}\right)=H{\left(U_{i}\right)}^{†}Y_{j} \end{array}$$

where $H^{†}\left(U_{i}\right)={\left[H{\left(U_{i}\right)}^{T}H\left(U_{i}\right)\right]}^{-1}H{\left(U_{i}\right)}^{T}$ denotes the Moore-Penrose pseudoinverse. Similarly,

(17) $$\begin{array}{l} {Â}_{j}^{b}=\underset{A_{j}^{b}}{\mathrm{argmin}}W\left(A_{j}^{b}\right)=H{\left(Y_{j}\right)}^{†}U_{i} \end{array}$$

The corresponding operations in MATLAB are given below.


for i=1:nu
   for j=1:ny
      HUij = hankel(U(1:end-nb,1),
         U(end-nb:end,1));
      bhat(i,j,:) = HUij\ Y(nb:end,j);
      W(i,j) = norm(Y(nb:end,j) -
         HUij^*^squeeze(bhat(i,j,:)));
   end
end


The backslash operator solves the LS problem in a numerically stable way using a QR factorization of the Hankel matrix. For model structure selection, that is, the problem of selecting the model order *n*_*b*_, the QR factorization enables all parameter estimates and cost functions for lower model orders to be obtained for free. However, model order selection is prone to overfitting; thus, in practice, one has to be careful when selecting *n*_*b*_ not only based on the LS cost function.

### 2.7. Regularization

Due to the challenge of avoiding overfitting, encoding and decoding techniques should be complemented with a regularization method, which basically adds a penalty for the model complexity to (15). In general terms, regularized LS can be expressed as

(18) $$\begin{array}{l} V_{N}\left(B_{i}^{f}\right)=W_{N}\left(B_{i}^{f}\right)+\lambda \text{g}\left(B_{i}^{f}\right) \end{array}$$

where *N* is the number of data and **g** is generally called a *regularizer* or *regularization function*, and it is typically non-smooth and possibly non-convex and λ ∈ IR^+^ is a penalty parameter. The regularization function is most commonly selected as the *l*_*p*_ norm, i.e.,

(19) $$\begin{array}{l} \underset{B_{i}^{f}}{\text{minimize}}\;\frac{1}{2}∥Y_{j}-H\left(U_{i}\right)B_{i}^{f}\;{∥}_{2}^{2}+\lambda ∥B_{i}^{f}{∥}_{p} \end{array}$$

With *l*_2_, the problem given in (19) has the analytic solution

(20) $$\begin{array}{l} {\hat{B}}_{i}^{f}={\left(H{\left(U_{i}\right)}^{T}H\left(U_{i}\right)+\lambda I\right)}^{-1}H{\left(U_{i}\right)}^{T}Y_{j} \end{array}$$

Similarly,

(21) $$\begin{array}{l} {Â}_{j}^{b}={\left(H{\left(Y_{j}\right)}^{T}H\left(Y_{j}\right)+\lambda I\right)}^{-1}H{\left(Y_{j}\right)}^{T}U_{i} \end{array}$$

However, *l*_2_ regularization does not do a variable subset selection.

Methods that directly aim to limit the number of parameters *n*_*b*_ include Akaike's information criterion AIC, where *U*_*N*_ = log(*W*_*N*_) + 2*n*_*b*_/*N*, and his improved suggestion Bayesian information criterion BIC *U*_*N*_ = log(*W*_*N*_) + log(*n*_*b*_)/*N*. Note that *n*_*b*_ is the *l*_0_ norm of $B_{i}^{f}$, a fact that is used in many recent approaches of sparse modeling based on efficient algorithms for convex optimization. However, the *l*_0_ term is not convex, but the *l*_1_ norm is, and it is in practice a good approximation of the *l*_0_ norm (Ramirez et al., 2013). This trick to obtain a feasible problem belongs to the class of convex relaxations.

The use of the *l*_1_ norm to induce sparsity is frequently referred to as the *least absolute shrinkage and selection operator (LASSO)* (Tibshirani, 1996). This formulation can be used to identify the sparse spatial-temporal resolution and reveal information about the listening attention.

Conceptually, sparse signal estimation depicts a signal as a sparse linear combination of active elements, where only a few elements in *B*_*i*_ are non-zero. The sparse estimation can be further improved with group sparsity, in other words, grouping the elements in $B_{i}^{f}$ (or $A_{j}^{b}$) and considering the groups of elements to be singletons, where a relatively small number of these groups is active at each time point. The group sparse estimation problem is frequently referred to as *group LASSO* (Yuan and Lin, 2006).

One way to solve sparse (*l*_1_-regularized) optimization problems is to apply the Expectation Maximization (EM) algorithm. One such example is the sparse (*l*_1_-regularized) recursive least squares (SPARLS) algorithm introduced in Babadi et al. (2010). The SPARLS algorithm estimates a sparse forward model using a dictionary of atoms, which is posed as a linear estimation problem. It has already been successfully used in AAD studies to estimate the encoding model (Akram et al., 2017). The authors concluded that the SPARLS algorithm could improve performances over the conventional (*l*_2_-regularized) linear estimation methods. Another way to solve sparse (*l*_1_-regularized) optimization problems is based on proximal splitting algorithms, one of which is a forward-backward splitting (FBS) algorithm, also referred to as the proximal gradient method (Combettes and Pesquet, 2011). Recently, Miran et al. (2018) suggested a Bayesian filtering approach for sparse estimation to tackle AAD. In their work, the authors used FBS procedure for decoding/encoding model estimation in real-time. In our examples, we use an algorithm called ADMM (alternating direction method of multipliers) to solve sparse (*l*_1_-regularized) optimization problems in an efficient way that normally requires very few iterations of simple computations to converge. The reason is 2-fold: the ADMM is simpler and easier to work with, since its iterative solution can be implemented via simple analytical expressions, and it has a proven fast convergence (Boyd et al., 2011).

### 2.8. SIMO Formulation

For simplicity, we have thus far considered single-input single-output SISO models, where the model relates one sound source to one EEG signal, and conversely for the reverse model. It is, however, simple to extend the model to a single-input multiple-output (SIMO) model that aims to explain all EEG data based on one sound stimulus at a time. The principle is that the sound stimulus that best explains the observed EEG signals should correspond to the attended source.

The SIMO FIR model for each sound source is defined as

(22) $$\begin{array}{l} Y=H\left(U_{i}\right)B_{i}^{f}+E_{i}^{f},\;i=1,2,\cdots \;,n_{u}, \end{array}$$

where $B_{i}^{f}$ is an *n*_*b*_ × *n*_*y*_ matrix.

In the literature, the filter ***B***_*i*_ is frequently referred to as a *temporal response function* (TRF), and the corresponding case for the backward approach leads to an *n*_*a*_ × *n*_*y*_ matrix ***A***^*b*^, where $A^{b}=vec\left(A_{j}^{b}\right)$, referred to as a *decoder*.

#### 2.8.1. Example 1

**Figure 2:**
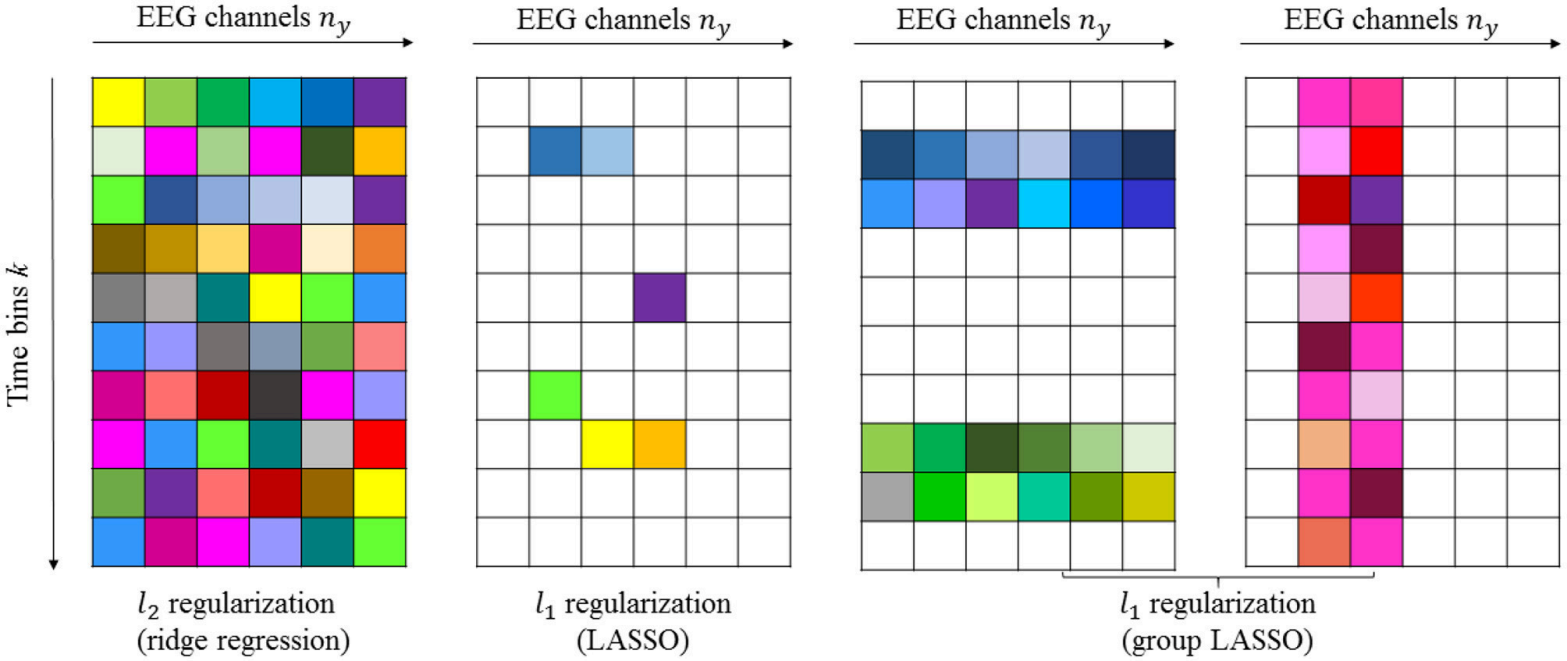
Schematic illustration of dense and sparse modeling. The first panel shows the “dense” filter resulting from *l*_2_-penalized LR. The second, third, and fourth panels show the sparse filters resulting from *l*_1_-penalized LR generated by LASSO and group LASSO. Non-active (zero-valued) elements are shown in white.

If we assume that *n*_*b*_ = 10 and *n*_*y*_ = 6, then we can estimate $\hat{B_{i}^{f}}$, as shown in Figure 2. The first panel in Figure 2 shows the “dense” filter ***B***_*i*_, where all the elements are active (non-zero). The second panel in the same figure illustrates the sparse matrix resulting from *LASSO*. Here, LASSO finds the active elements in the filter $B_{i}^{f}$ (elements in white are non-active or zero-valued elements). The prior knowledge of how the time lags and electrodes form the groups can be incorporated with group LASSO to obtain filters similar to those in the last two panels shown in Figure 2, respectively. If for instance some of the EEG signals are completely uncorrelated with the sound stimulus, the reconstruction error will not increase if these EEG signals are left out. A general rule of thumb for intuition in system identification is that zero is the best prediction of zero mean white noise. Any other prediction will increase the cost. That is the rationale with LASSO, don't attempt to predict white noise, even if reasons of over learning may indicate that it is possible.

### 2.9. CCA vs. Linear FIR Filters

The main difference between the forward and backward models is how the noise enters the models 6 and 7, respectively. The general rule in LS estimation is that the noise should be additive in the model. If this is not the case, then the result will be biased. However, if there is additive noise to both the input *U*_*i*_ and the output *Y*_*j*_, then the total least squares (TLS) algorithm can be used. TLS basically weights both noise sources together in an optimal way. The standard implementation of TLS is based on a singular value decomposition (SVD) of the Hankel matrix $H\left(U_{i}\right)$.

CCA combines the encoding and decoding approaches:

(23) $$\begin{array}{l} B_{i}^{f}\left(q\right)u_{i}\left[k\right]\sim \overset{n_{y}}{\underset{j=1}{\sum }}A_{j}^{b}\left(q\right)y_{j}\left[k\right]+e\left[k\right]\;\left(\text{CCA}\right) \end{array}$$

and involves *solving a generalized eigenvalue problem*.

Table 1 provides a summary of the discussed linear models.

**Table 1:** An overview of linear methods.

**Learning representations**	**Approaches**	**Mathematical formulations**	**Optimization problem**	**Relevant references**
Correlation- based learning	*Cross-Correlation* CCA	$B_{i}^{f}\left(q\right)u_{i}\left[k\right]\sim \overset{n_{y}}{\underset{j=1}{\sum }}A_{j}^{b}\left(q\right)y_{j}\left[k\right]+e\left[k\right]$	Generalized eigenvalue problem	Biesmans et al., 2017; Dmochowski et al., 2017; de Cheveigné et al., 2018; de Cheveigné et al., 2019
Model-based learning	Forward modeling Supervised case: *Encoding*	$y_{j}\left[k\right]=B_{i}^{f}\left(q\right)u_{i}\left[k\right]+e_{j}^{f}\left[k\right]$	Least- squares	Ding and Simon, 2012a; Di Liberto et al., 2015; Alickovic et al., 2016, in rewiev; Fiedler et al., 2017, 2019; Hjortkjær et al., 2018; Kalashnikova et al., 2018; Lesenfants et al., 2018; Lunner et al., 2018; Verschueren et al., 2018; Wong et al., 2018
	Inverse/backward modeling Supervised case: *Decoding*	$u_{i}\left[k\right]=\overset{n_{y}}{\underset{j=1}{\sum }}A_{j}^{b}\left(q\right)y_{j}\left[k\right]+e_{i}^{b}\left[k\right]$		Mirkovic et al., 2015; O'Sullivan et al., 2015, 2017; Aroudi et al., 2016; Das et al., 2016, 2018; Presacco et al., 2016; Biesmans et al., 2017; Fuglsang et al., 2017; Van Eyndhoven et al., 2017; Zink et al., 2017; Bednar and Lalor, 2018; Ciccarelli et al., 2018; Etard et al., 2018; Hausfeld et al., 2018; Narayanan and Bertrand, 2018; Schäfer et al., 2018; Vanthornhout et al., 2018; Verschueren et al., 2018; Wong et al., 2018; Akbari et al., 2019; Somers et al., 2019

Solving a generalized eigenvalue problem is more costly for high-dimensional data in a computational sense (Watkins, 2004). In particular, the sample covariance matrices of high-dimensional data become singular (do not have an inverse), which leads to more complex associated generalized eigenvalue problems.

A regularized CCA (rCCA) is often proposed to address this problem (Hardoon et al., 2004). This particular problem may be overcome by formulating CCA as an LS problem, as in Sun et al. (2011), where the classical CCA (and rCCA) is formulated as an LS problem, and LS optimization methods are used to solve it. However, this topic is beyond the scope of this paper and is left for future work.

### 2.10. Non-linear Models

Linear models should always be examined first in the spirit of “try simple things first.” An alternative method to estimate the attended sound source would be to exploit non-linear models. There are, however, many problems in ML that require non-linear models. The principle is the same, but the algorithms are more complex. In short, the linear model $Y_{j}=H\left(U_{i}\right)B_{i}+E_{j}$ in (12) is replaced with

(24) $$\begin{array}{l} Y_{j}=f\left(U_{i},B_{i}\right)+E_{j}. \end{array}$$

Among the standard model structures for the non-linear function *f*, we mention the Wiener and Hammerstein models, support vector machines and neural networks (Taillez et al., 2017; Deckers et al., 2018; Akbari et al., 2019). Indeed, non-linear models can be used to decipher attention, but the focus of this paper is on linear models because they are simpler to understand and implement.

## 3. Examined Datasets

We have used both simulated data and real datasets to evaluate the aforementioned algorithms. Simulations provide a simple way to test, understand and analyze complex algorithms in general, as well as in this case. We use synthetic sound and EEG signals to illustrate the aforementioned algorithms, but real data have to be used to evaluate the potential for applications.

In our contribution, we are revisiting two datasets that were anonymized and publicly available upon request by the previous authors. The publications from which the data originated (see references Power et al., 2012; Fuglsang et al., 2017) state that the data were collected with the approval of the corresponding ethical bodies and with due process of informed consent.

The *first real dataset* is characterized as follows:

- The subjects were asked to attend to a sound source on either the left *u*_1_ or the right *u*_2_ side.
- The subjects maintained their attention on one sound source throughout the experiment.
- Each subject undertook 30 trials, each 1 min long.
- Each subject was presented with two works of classic fiction narrated in English in the left and right ears.
- Full-scalp EEG data were collected at a sampling frequency of 512 Hz with *n*_*y*_ = 128 number of electrodes.
- Sound data were presented at a sampling frequency of 44.1 kHz.

This dataset was first presented and analyzed in Power et al. (2012) and O'Sullivan et al. (2015). Henceforth, we refer to this dataset as the *O'Sullivan dataset*.

The *second dataset* can be described as follows:

- The subjects were asked to *selectively* attend to a sound source on the left *u*_1_ or right *u*_2_ side in different simulated acoustic environments (anechoic, mildly reverberant classroom, and highly reverberant Hagia Irene Church) throughout the experiment.
- The subjects switched their attention from one sound source to another throughout the experiment.
- Each subject was presented with two works of classic fiction narrated in Danish.
- Each subject undertook 60 trials, each 50 s long accompanied by multiple choice questions.
- Full-scalp EEG data were collected at a sampling frequency of 512 Hz with *n*_*y*_ = 64 number of electrodes.
- Sound data were presented at a sampling frequency of 44.1 kHz.

This dataset was first presented and analyzed in Fuglsang et al. (2017), and we will refer to this dataset as the *DTU dataset*.

We randomly selected twelve subjects from each dataset to assess the potential benefits that might result from the different linear models considered in this contribution. The reason for this approach is that our main contribution is to provide a tutorial of methods and examples of their use, not to obtain a final recommendation on which method is the best in general.

There are several toolboxes that are useful when working with real datasets. First, there are at least two toolboxes available for loading EEG data: (1) the EEGLab toolbox (https://sccn.ucsd.edu/eeglab/) (Delorme and Makeig, 2004) and (2) the FieldTrip toolbox (http://www.fieldtriptoolbox.org/) (Oostenveld et al., 2011). For more details on importing EEG data with EEGLab and FieldTrip, see Appendix. Then, linear trends can be removed, and the EEG data can be normalized using functions in the NoiseTools toolbox (de Cheveigné and Simon, 2008a,b; de Cheveigné, 2010, 2016).

## 4. Computational Models in Practice

In this section, we apply the presented algorithms to the two datasets described in Section 3. All experiments were performed on a personal computer with an Intel Core(TM) i7 2.6 GHz processor and 16 GB of memory, using MATLAB R2015b. Note that for notational simplicity we shall take ***A***^*b*^ = ***A*** and $B_{i}^{f}=B_{i}$ in the remainder of this section.

We start by discussing two main alternatives to train the models and estimate the de/en - coders (***A*** or ***B***):

1. Treating each trial as a single least-squares LS problem and estimating one de/en-coder for each training trial separately, and averaging over all training de/en-coders (Crosse et al., 2016).
  (25) $$\begin{array}{l} B_{i}^{avg}=1/K\underset{k}{\sum }\left[{\left(H{\left(U_{i,k}\right)}^{T}H\left(U_{i,k}\right)\right)}^{-1}H{\left(U_{i,k}\right)}^{T}Y_{j,k}\right] \end{array}$$
2. Concatenating all training trials in a single LS problem (Biesmans et al., 2017).
  (26) $$\begin{array}{l} B_{i}^{conc}={\left[\underset{k}{\sum }H{\left(U_{i,k}\right)}^{T}H\left(U_{i,k}\right)\right]}^{-1}\left[\underset{k}{\sum }H{\left(U_{i,k}\right)}^{T}Y_{j,k}\right] \end{array}$$

Here *K* is a total number of trials. We may point to the following aspects that are to be considered when discussing the two alternatives:

- *Averaging LS per-trial estimates is not equivalent with the correct overall LS estimate*. It is easy to show that the two alternatives will result in different estimates, even if the discontinuities and boundary effects are correctly treated. One can show algebraically that -under some technical conditions- the second alternative will yield a better estimator with a lower (co-)variance on its entries. For a more detailed discussion, see section 2.2.1 in Gustafsson (2010).
- *Efficient cross-validation*. Note that the matrix $H{\left(U_{i}\right)}^{T}H\left(U_{i}\right)$ in (20) denotes the information matrix, and can also be expressed as $\underset{k}{\sum }H{\left(U_{i,k}\right)}^{T}H\left(U_{i,k}\right)$, where *k* is a trial index and *U*_*i,k*_ contains the data from one trial. This trick of combining sufficient statistics for the different datasets saves a lot of computations. For a more detailed discussion, see sections 2.2.3, 2.2.4 in Gustafsson (2010).
- *Introducing artifacts from discontinuities between trials*. The issue of introducing artifacts from discontinuities between trials is due to the boundary effects when the filter shifts out of the window. One solution is to insert zeros in the Hankel matrix used for solving the LS problem. A better alternative is to delete the rows in the Hankel matrix affected by these boundaries, which yields an LS estimate without boundary effects. In a similar way, one can remove discontinuities between trials in the concatenation case. For more details, see section 6.3 in Gustafsson et al. (2010).

Although both alternatives have been widely used as tools for studying selective attention and AAD, we shall here consider the first alternative. A basic reason for this is that the first alternative has received somewhat more attention in the literature due primarily to being implemented in the publicly available mTRF toolbox. It is also important to note that the second alternative is often less sensitive to the choice of the regularization parameter, and for which regularization can sometimes even be omitted if sufficient data is available (Biesmans et al., 2017).

### 4.1. Canonical Correlation Analysis

We start by evaluating the CCA model. The simple CCA model consists of the following steps:

- Design a multichannel representation of the input sound signal, e.g., cochlear or any other auditory model, time-frequency analysis with spectrogram, or Mel-frequency cepstral coefficients (MFCC) (Slaney, 1998).
- Demand two linear transformations with CCA. Efficient CCA-based decoding implementations are available in (1) COCOHA toolbox (https://cocoha.org/the-cocoha-matlab-toolbox/), (2) NoiseTools toolbox, (3) http://www.imt.liu.se/~magnus/cca/ and (4) http://www.yelab.net/software/CCA/. A particularly simple way of implementing CCA is available in MATLAB 's *canoncorr.m* function. This function takes Hankel matrices $H\left(U_{i}\right)$ and H(Y) with time lags [defined as in (4)] as inputs and computes the filters ***A***, ***B***_*i*_ and correlation coefficients (Krzanowski, 2000).
- Select the first (few) component(s) for each transformation such that the highest possible correlation between the datasets is retrieved.

#### 4.1.1. Example 2 (Attention Deciphering With CCA)

In this example, we consider one (randomly selected) subject from the first database who attended to the speech on his left side *U*_1_. The task is to determine whether CCA can be used to identify whether the attended speech is actually *U*_1_.

##### 4.1.1.1. Preprocessing

We followed the very simple preprocessing scheme described in the last sentence of §2.1 and in Alickovic et al. (2016).

##### 4.1.1.2. Modeling

Following the approach to CCA proposed here, see Equation (23), the encoding and decoding filters covered time lags ranging from −250 ms to 0 ms prestimulus (see Alickovic et al., 2016) and 0 ms to 250 ms poststimulus (see O'Sullivan et al., 2015), respectively.

##### 4.1.1.3. Classification

After projecting data onto a lower-dimensional space, a linear SVM is applied for binary classification: attended vs. ignored sound. We select the correlation coefficient values as the classifier's inputs. In this example, we selected the first 10 coefficients, thus classifying two times with a 10-D vector, once for the attended sound and once for the ignored sound. This corresponds to a 2-fold match-mismatch classification scheme suggested in de Cheveigné et al. (2018). In the case that the classifier implies attention on both sounds (attended and ignored), we consider such classification as incorrect. Next, we generate 10 random partitions, i.e., 10-fold cross-validation (CV), of data into training (27 minutes) and test (3 minutes) sets, and we report the average performances.

##### 4.1.1.4. Results

The average classification accuracy is ~ 98%. The total computational time for training and CV is ~ 20 s.

##### 4.1.1.5. Remarks

Note that this accuracy could be further improved with more training data or further preprocessing (e.g., removing eye blinks from EEG data). However, because we aim to establish real-time systems, we attempt to reduce the preprocessing and thereby increase the speed of the system at the expense of a lower accuracy rate.

As for any data-driven model design, the choice of the classifier's inputs is left to the user. Our choice is based primarily on the desire to show that CCA is a promising tool for auditory attention classification. In the following sections, we further discuss the significance of CCA by comparing the results of the methods discussed here applied on the two large datasets described in section 3.

### 4.2. Decoding With Dense Estimation

SR is the most prominent decoding technique, see Equation (11), that aims to reconstruct the stimuli from the measured neural responses. The standard approach to SR in the literature is to use *l*_2_-regularized (dense) LR techniques. The recent work of Crosse et al. (2016) provides a comprehensive description of the Multivariate Temporal Response Function (mTRF) toolbox (https://sourceforge.net/projects/aespa/)—a MATLAB toolbox for computing (dense) filters *A*_*j*_ or *B*_*i*_ (depending on a mapping direction) by using LR techniques.

#### 4.2.1. Example 3 (Attention Deciphering With Dense SR)

Here, we consider the same subject as in the previous example. The task is now to determine the efficiency of the dense SR in classifying the attended speech.

##### 4.2.1.1. Preprocessing

Identical to Example (4.1.1).

##### 4.2.1.2. Modeling

The decoder ***A*** covers time lags up to 250 ms poststimulus. To find the decoder ***A***, the model presented in Equation (11) is applied. One decoder is produced for each stream of sound *i* for each segment *s* = 1, …, 30, resulting in 30 attended decoders.

##### 4.2.1.3. Classification

Next, 29 of these decoders are combined by simply averaging ***A*** matrices to the matrix ***A***_*avg*_ in the training phase - LOOCV (leave-one-out CV); then, ***A***_*avg*_ is used to produce the estimate of the stimulus Û_*i*_ for the fresh data, i.e., the remaining segment. The correlation coefficient *c* is then assessed between the actual *n*_*u*_ test stimuli *U*_*i*_ and the estimate Û_*i*_, and the sound stream with the greatest *c* is identified as the attended source. This procedure is repeated 30 times.

##### 4.2.1.4. Results

The average classification accuracy is ~ 80%. Note the drop in accuracy from ~ 98% (obtained with CCA) to ~ 80% (with SR) for this particular subject. The total computational time for training and CV is ~ 58 s.

### 4.3. Decoding With Sparse Estimation

In this section, we consider SR, but we use *l*_1_ (sparse) regularization rather than *l*_2_ (dense) regularization (which is widely used in auditory research) to quantify the sparsity effect on the auditory attention classification.

#### 4.3.1. Example 4 (Attention Deciphering With Sparse SR)

Using the data from the same subject as in Examples (4.1.1–4.2.1), the task is to evaluate the performances of *l*_1_-regularized (sparse) SR.

##### 4.3.1.1. Preprocessing

*4.3.1.1.1. Preprocessing/Modeling/Classification* Identical to Example (4.1.1).

##### 4.3.1.2. Preprocessing

*4.3.1.2.1. Results* The average classification accuracy is ~ 80%. The total computational time for training and CV is ~ 6 s. Note the drop in computational time from ~ 58 s (obtained with dense SR) to ~ 6 s (obtained with sparse SR) for this particular subject.

##### 4.3.1.3. Preprocessing

*4.3.1.3.1. Remarks* Note the substantial reduction in the computational time when *l*_1_ regularization, implemented with the ADMM, is used rather than conventional *l*_2_ regularization in the SR method.

### 4.4. Encoding With Dense Estimation

Here, we consider encoding, where we go in the forward direction from the speech to EEG data. The standard approach to encoding found in the auditory literature is to solve the optimization problem (10) for each EEG channel *j* = 1, …, *n*_*y*_ separately, which means that we will have *n*_*y*_ neural predictions for each stimulus. Recall that one single reconstruction for each stimulus in the decoding approach discussed above makes it easier to compare the correlation coefficient values (CCVs). One way to classify the attended sound source by using the encoding approach is to take the sum of all CCVs, compare these sums, and classify the attended sound as the one with the highest sum of the CCVs (similar to the decoding). We refer to this approach as *dense LOOCV encoding*.

#### 4.4.1. Example 5 (Attention Deciphering With Dense LOOCV Encoding)

Here, we consider the same subject as in the previous examples. The task is now to determine the efficiency of the suggested approach to dense encoding in classifying the attended speech.

##### 4.4.1.1. Preprocessing

Identical to Example (4.1.1).

##### 4.4.1.2. Modeling

The TRF ***B***_*i*_ covers time lags from -250 ms to 0 ms prestimulus. To find the TRF ***B***_*i*_, the model presented in Equation (10) is applied. One TRF is produced for each stream of sound *i* for each segment *s* = 1, …, 30, resulting in 30 attended TRFs.

##### 4.4.1.3. Classification

Next, 29 of these TRFs are combined by simply averaging ***B***_*i*_ matrices to the matrix ***B***_*i,avg*_ in the training phase - LOOCV (leave-one-out CV); then, ***B***_*i,avg*_ is used to predict the neural response Ŷ_*i*_ for the fresh data, i.e., the remaining segment. The summed CCV is then assessed between the actual *Y* and predicted Ŷ_*i*_, and the sound stream with the larger CCV is identified as the attended source, i.e.,

(27) $$\begin{array}{l} î=\arg\underset{i}{\max}CCV_{i} \end{array}$$

This procedure is repeated 30 times.

##### 4.4.1.4. Results

The average classification accuracy is ~ 77%. The total computational time for training and CV is ~ 2.5 s. However, the main limitation of the dense encoding is that it is very sensitive to the regularization parameter λ, which must be selected very carefully. We will return to this issue in section 4.7.

##### 4.4.1.5. Remarks

Note the substantial reduction in the computational time with dense encoding compared to the dense decoding (SR) method.

### 4.5. Encoding With Sparse Estimation

Here, we consider encoding with ADMM-based sparse estimation. We report similar performance in terms of both the classification accuracy rate and computational time as observed for the encoding with dense estimation for the data taken from the same subject used in the previous examples. We refer to this approach as *sparse LOOCV encoding*.

#### 4.5.1. Example 6 (Attention Deciphering With Sparse LOOCV Encoding

Here, we consider the same subject as in the previous examples. The task is now to determine the efficiency of the suggested approach to sparse LOOCV encoding in classifying the attended speech.

##### 4.5.1.1. Preprocessing, Modeling & Classification

As in Example (4.4.1).

##### 4.5.1.2. Results

The average classification accuracy is ~ 80%. The total computational time for training and CV is ~ 1.5 s. Note that LOOCV encoding could be quite sensitive to λ.

### 4.6. Encoding From the System Identification Perspective

Here, we take a different approach to the common classification approaches found in the auditory literature, using tools from the system identification area (Ljung, 1998). In the present work, we refer to this approach as *adaptive encoding*.

#### 4.6.1. Example 7 (Attention Deciphering With the SI Approach)

We consider the same data used in our previous examples. The task is now to use our classification model.

##### 4.6.1.1. Preprocessing

Identical to Example (4.1.1).

##### 4.6.1.2. Modeling

The TRF *B*_*i*_ covers time lags from −250 ms to 0 ms prestimulus. The attended and ignored TRFs ***B***_1_ and ***B***_2_ are computed for each segment, and the cost for both TRFs is evaluated for each segment as Lunner et al. (2018)

(28) $$\begin{array}{l} V_{i}\left(B_{i}\right)=∥Y-U_{i}B_{i}{∥}_{F}^{2}+\lambda ∥{\bar{B}}_{i}{∥}_{1} \end{array}$$

(29) $$\begin{array}{l} \text{subject to }B_{i}={\bar{B}}_{i} \end{array}$$

##### 4.6.1.3. Classification

We compare the costs for each segment and determine which speech signal provides the smallest cost, i.e.,

(30) $$\begin{array}{l} î=\arg\underset{i}{\min}V_{i}\left(B_{i}\right) \end{array}$$

If λ is known a priori, then this model is unsupervised and requires no training. However, this is rarely the case, and λ must be computed separately for each subject by using the subject's own training data.

##### 4.6.1.4. Results

We use the first 9 min of data to compute the value of the regularization parameter λ and the remaining time to assess the performances of the models given in (28)-(30). The average classification accuracy is ~ 95%.

##### 4.6.1.5. Remarks

Although the classification accuracy of the adaptive encoding approach is similar to that obtained with CCA, note the substantial decrease in training time, from 27 to only 9 min.

### 4.7. Sensitivity of the Regularization Parameter

The previously discussed models have all been sensitive to a regularization parameter λ. Therefore, we need to solve the optimization problem (19) for different λ values to identify the λ value that optimizes the mapping such that the optimal λ value minimizes the mean squared error (MSE) and maximizes the correlation between the predicted (reconstructed) and actual waveform. One way to perform this optimization is to have the inner CV loop on the training data to tune λ value. In the inner CV loop, we can implement either LOOCV or *K*-fold CV in a similar way to the outer LOOCV, with the difference that we repeat the process for different λ values and select the λ that yields either the lowest MSE or the highest correlation (Pearson *r*) value. For the *l*_2_ (dense) regularization, a parameter sweep is generally performed between 10^−6^ and 10^8^ (Wong et al., 2018). From our experience, a good choice for this type of regularization is to set λ to 10^3^. For the *l*_1_ (sparse) regularization, the parameter sweep is typically performed between $10^{-6}\lambda_{max}$ and 0.95λ_*max*_, where λ_*max*_ is a critical value above which the filter becomes zero-valued (Boyd et al., 2011). From our experience, a good choice for this type of regularization is to set λ to $10^{-1}\lambda_{max}$. A similar approach was adapted for the adaptive encoding, with the only difference that the inner CV loop was implemented on 9 min of data.

### 4.8. Classification Performance Comparison

In this section, we verify that the proposed linear models discussed in the present contribution can identify the sound source of the listener's interest. Two different datasets, the O'Sullivan and DTU datasets, were used to evaluate the performances of different models. Here the window length over which the correlation coefficients are estimated for each method is the same as in the corresponding examples above and the trial lengths are the same as the trial lengths mentioned in section 3.

#### 4.8.1. O'Sullivan Dataset

Table 2 shows part of the assessed performances when the subjects were asked to attend to an identical sound source throughout the experiment. As shown in this table, CCA and adaptive encoding approaches resulted in the highest classification rates and the lowest computational times (see the previous examples). Moreover, note that the sparse estimation outperformed the dense estimation for both SR and LOOCV encoding. The accuracy rates for sparse SR were ~5% higher, on average, when sparse (ADMM-based) estimation was used to determine the (decoder) filter coefficients. This was also the case when estimating the encoding filter coefficients. Furthermore, there was a significant reduction in computational time, as shown in Table 3. Although it might seem natural that *l*_2_ regularization would be faster as *l*_1_ regularization is iterative process, what makes *l*_1_ regularization faster is the ADMM algorithm that converges quickly enough, within few iteration steps and does not include inverting large matrices.

**Table 2:** Classification rates on the O'Sullivan dataset for the different classification approaches discussed in this contribution.

	**Subject**	**Dense SR**	**Sparse SR**	**Dense LOOCV encoding**	**Sparse LOOCV encoding**	**Adaptive encoding**	**CCA**
Attend Right	1	86.21	93.10	86.21	89.66	100	97.86
	2	86.67	90.00	70.00	70.00	95.45	98.32
	3	96.67	100.00	86.67	86.67	100.00	97.93
	4	90.00	90.00	80.00	76.67	86.36	98.33
	5	90.00	96.67	90.00	93.33	95.45	98.03
	6	70.00	86.67	60.00	70.00	100.00	97.83
	Avg	86.59	92.74	78.81	81.05	96.21	98.05
Attend Left	7	80.00	86.67	63.33	73.33	100.00	98.33
	8	93.33	90.00	76.67	80.00	95.45	97.70
	9	80.00	80.00	73.33	73.33	95.45	97.08
	10	80.00	90.00	73.33	76.67	81.82	96.90
	11	76.67	80.00	66.67	83.33	95.45	98.25
	12	100.00	100.00	83.33	86.67	100.00	98.32
	Avg	85.00	87.78	72.78	78.89	94.70	97.76
Total avg		85.80	90.26	75.80	79.97	95.45	97.91

**Table 3:** Computational times on the O'Sullivan dataset for the different classification approaches discussed in this contribution.

	**Subject**	**Dense SR**	**Sparse SR**	**Dense LOOCV encoding**	**Sparse LOOCV encoding**	**Adaptive encoding**	**CCA**
Attend Right	1	46.69	5.21	2.06	1.99	1.96	23.34
	2	47.65	2.20	2.09	86.67	2.05	23.73
	3	49.44	2.20	2.38	76.67	2.38	20.75
	4	47.98	2.20	2.55	93.33	2.45	19.83
	5	47.95	2.20	2.09	70.00	2.00	19.58
	6	47.75	2.17	2.56	70.00	2.36	27.83
	Avg	47.91	5.43	2.17	2.28	2.20	22.51
Attend Left	7	47.61	5.26	2.16	2.20	2.15	20.32
	8	42.34	6.08	2.19	2.16	2.12	21.19
	9	43.03	5.28	2.15	2.08	2.06	19.53
	10	44.79	6.26	2.18	2.45	2.37	19.82
	11	43.30	5.28	2.19	2.14	2.10	19.91
	12	49.73	5.29	2.22	2.04	2.01	21.19
	Avg	45.13	5.57	2.18	2.18	2.08	20.33
Total avg		46.52	5.50	2.18	2.23	2.13	2.16

As shown in Tables 2, 3, the best-performing linear methods for this dataset in terms of both accuracy and computational time are *adaptive encoding* and *CCA*.

#### 4.8.2. DTU Dataset

Table 4 shows part of the assessed performances when the subjects were asked to switch their attention throughout the experiment. As shown, CCA results in the highest classification rates. Moreover, note that for this dataset, the sparse estimation also outperformed the dense estimation for both SR and LOOCV encoding. However, the adaptive encoding did not result in a high classification accuracy rate for the “switching” data compared to CCA. One reason for this result might be that CCA, as a “bidirectional” approach, captures more of the EEG-audio (stimulus-response) data relationship than when going in only one (forward) direction. To summarize, all linear methods have a high potential to be fully utilized in the identification of the subject's sound source of interest in “attention-switching scenarios,” with CCA demonstrating a high potential to also be used as an efficient AAD tool.

**Table 4:** Classification rates on the DTU dataset for the different classification approaches discussed in this contribution.

**Subject**	**Dense SR**	**Sparse SR**	**Dense LOOCV encoding**	**Sparse LOOCV encoding**	**Adaptive encoding**	**CCA**
1	83.33	83.33	71.67	71.67	80.39	87.23
2	78.33	90.00	78.33	76.67	70.59	81.93
3	86.67	81.67	66.67	73.33	86.27	80.73
4	90.00	96.67	70.00	66.67	78.43	98.75
5	81.67	81.67	75.00	60.00	70.59	82.90
6	70.00	73.33	68.33	71.67	84.31	100.0
7	76.67	80.00	78.33	78.33	80.39	94.63
8	91.67	93.33	71.67	73.33	70.59	81.08
9	81.67	85.00	80.00	75.00	80.39	97.97
10	85.00	88.33	70.00	75.00	84.31	96.18
11	91.67	90.00	60.00	73.33	78.43	82.54
12	88.33	88.33	63.33	66.67	80.72	85.77
Total avg	83.75	85.97	71.11	72.22	78.33	89.14

The O'Sullivan dataset is known to be biased in the sense that subjects either always maintain their attention on the left sound source or always maintain their attention on the right sound source. The subject-dependent decoders then tend to perform much better than when they are trained on both left- and right- attended trials of the same subject. This effect was shown in Das et al. (2016). This partially explains why the performance on the DTU dataset is noticeably lower.

It is, however, important to keep in mind that although the tables above may indicate different performance among the methods, no comparative conclusions can be drawn from these tables, since the parameter settings may not be fully optimized or comparable. It is not the purpose of the paper to make that performance comparison, and rather just illustrate the different working principles. To objectively compare methods, one should use the same cross-validation, same window lengths to make a decision, and then properly optimize all parameters for each method.

## 5. Conclusions

In this work, we investigated the similarities and differences between different linear modeling philosophies: (1) the classical correlation-based approach (CCA), (2) encoding/decoding models based on dense estimation, and (3) (adaptive) encoding/decoding models based on sparse estimation. We described the complete signal processing chain, from sampled audio and EEG data, through preprocessing, to model estimation and evaluation. The necessary mathematical background was described, as well as MATLAB code for each step, with the intention that the reader should be able to both understand the mathematical foundations in the signal and systems areas and implement the methods. We illustrated the methods on both simulated data and an extract of patient data from two publicly available datasets, which have been previously examined in the literature. We have discussed the advantages and disadvantages of each method, and we have indicated their performance on the datasets. These examples are to be considered as inconclusive illustrations rather than a recommendation of which method is best in practice.

Furthermore, we presented a complete, step-by-step pipeline on how to approach identifying the attended sound source in a cocktail party environment from raw electrophysiological data.

## Author Contributions

All authors designed the study, discussed the results and implications, and wrote and commented the manuscript at all stages.

### Conflict of Interest Statement

The authors declare that the research was conducted in the absence of any commercial or financial relationships that could be construed as a potential conflict of interest.

## Abbreviations

AAD: auditory attention deciphering
ADMM: alternating direction method of multipliers
AIC: Akaike–s information criterion
BIC: Bayesian information criterion
CASA: computational auditory scene analysis
CCA: canonical correlation analysis
CCV: correlation coefficient value
CV: cross-validation
EEG: electroencephalography
FBS: forward-backward splitting
FIR: finite impulse response
IIR: infinite impulse response
LASSO: least absolute shrinkage and selection operator
LOOCV: leave-one-out cross-validation
LS: least squares
MEG: magnetoencephalography
MFCC: Mel-frequency cepstral coefficients
ML: machine learning
MSE: mean squared error
SIMO: single input multiple output
SISO: single input single output
SPARLS: sparse recursive least squares
SR: stimulus reconstruction
SVD: singular value decomposition
SVM: support vector machine
TLS: total least squares
TRF: temporal response function.

## Appendix: EEG Data Import

### A.1. Importing EEG Data With EEGLab

The key steps are as follows:

- Downloading the EEGLab toolbox.
- Starting MATLAB and adding the path.
- Loading the EEG data with the *pop_biosig* function.
- Excluding all non-scalp channels and reference to average all scalp channels as: *EEG = pop_select( EEG,'nochannel', 'channel names'); EEG = pop_reref( EEG, []);*
- Segmenting data correctly based on the trigger information with the *pop_epoch* function.
- Additionally, mean baseline value from each epoch can be removed with the *pop_rmbase* function.
- Saving the .mat file

### A.2. Importing EEG Data With FieldTrip

The key steps are as follows:

- Downloading the FieldTrip toolbox.
- Starting MATLAB and adding the path.
- Using the *ft_defaults* function to configure default variable and path settings.
- Reading the EEG data with a *ft_read_data* function to a structure file and adding the needed values to the fields in the structure from the header with the function *ft_read_header*.
- Reading the event information if possible with *ft_read_event*.
- Segmenting the data correctly based on the relevant event(s).
- Selecting the scalp channels.
- Removing the mean and normalizing the data.
